# Endoscopic Treatment for Nonhypertrophic Idiopathic Pyloric Stenosis in an Adolescent Patient

**DOI:** 10.1055/s-0043-57040

**Published:** 2023-05-03

**Authors:** Carlo Maria Ferlini, Elena De Lorenzi, Cristina Belgiovine, Emanuele Cereda, Silvia Maria Elena Caimmi, Alessandro Raffaele

**Affiliations:** 1 Pediatric Surgery Unit, Department of Maternal and Child Health, Fondazione IRCCS Policlinico San Matteo, Pavia, Lombardia, Italy; 2 Department of Clinical, Surgical, Diagnostics and Pediatric Sciences, University of Pavia, Pavia, Lombardia, Italy; 3 Clinical Nutrition and Dietetics Unit, Department of Oncology and Hematology, Fondazione IRCCS Policlinico San Matteo, Pavia, Lombardia, Italy; 4 Pediatrics Unit, Department of Maternal and Child Health, Fondazione IRCCS Policlinico San Matteo, Pavia, Lombardia, Italy

**Keywords:** nonhypertrophic, idiopathic, pyloric, stenosis, endoscopic, dilation, multidisciplinary approach

## Abstract

Nonhypertrophic idiopathic pyloric stenosis (NHIPS) is a rare occurrence in children. It could be related to peptic ulcers, but a definitive cause is yet to be found. Treatment is a matter of debate, ranging from medical to surgical. We report the case of a 15-year-old boy suffering postprandial vomiting and weight loss in the previous 3 months. NHIPS was diagnosed and successfully treated with several sessions of endoscopic pyloric dilation and jejunal feeding. In association with a multidisciplinary approach, endoscopic dilation should be considered as a first-line treatment to avoid surgery.

## Introduction

Nonhypertrophic idiopathic pyloric stenosis (NHIPS) has rarely been reported in the pediatric literature. It represents one of the possible causes of acquired gastric outlet obstruction together with other conditions of peptic, oncological, infectious, or caustic etiology. It must be suspected in patients older than 3 months (time limit associated with hypertrophic pyloric stenosis) 1 presenting recurrent postprandial vomiting and weight loss. This is especially true if no clear causes are evidenced by proper laboratory testing and imaging. Its causes are still a matter of debate. On occasions, it could be a possible sequela of previous peptic ulcers, 2 but in most reported cases, the ultimate cause remained unknown. There is no consensus on treatment options as well. Medical treatment with proton pump inhibitors (PPIs) is considered valid mainly for control of the possible underlying peptic origin, but insufficient per se. On the other hand, endoscopic pyloric dilation, repeated in several sessions, has been described as successful. 3 Finally, surgical procedures are reserved in case of medical and endoscopic treatment failure. Here, we present the case of an adolescent male with a history of chronic vomiting secondary to NHIPS managed with endoscopic dilations and jejunal feeding.

## Case Report

**Fig. 1:**
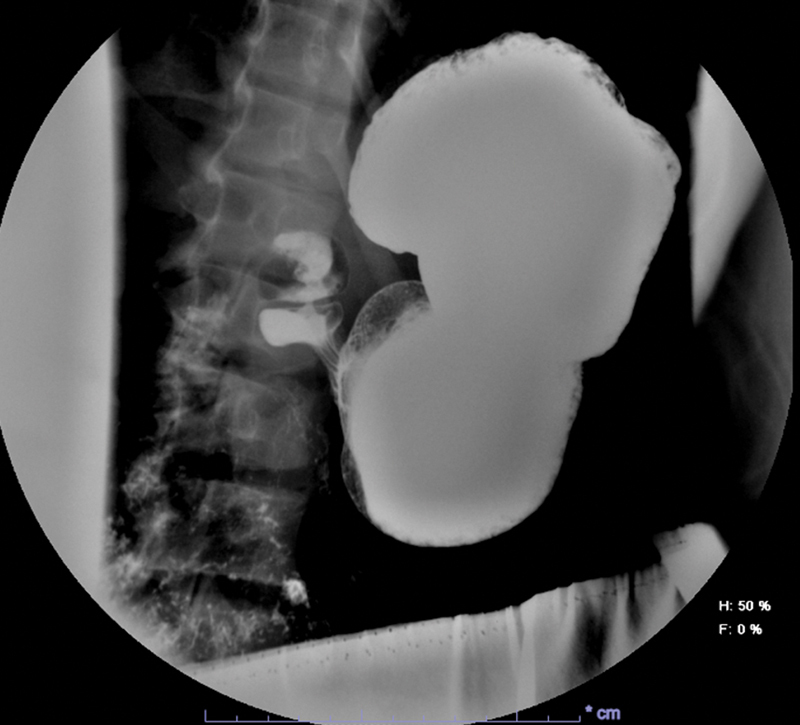
Upper gastrointestinal series showing oblong stomach and impaired pyloric transit (image taken 13 minutes after contrast instillation, age 15 years).

**Fig. 2:**
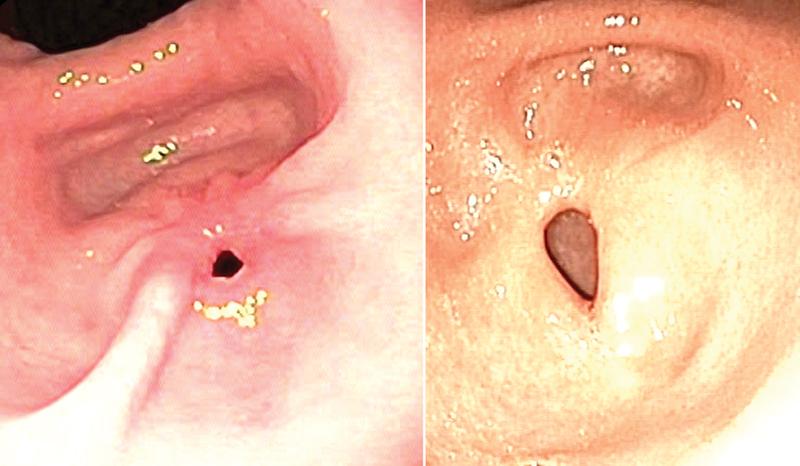
Endoscopic photographs of the pylorus at first esophagogastroduodenoscopy (EGD) (left) and at control EGD following all dilation sessions (right) (age 15 years).

A 15-year-old boy came to our attention with a complaint of postprandial epigastric pain, vomiting, and weight loss (6 kg) in the previous 3 months. His medical history was notable for growth retardation due to celiac disease diagnosed at the age of 9 years (subsequently regressed with dietary therapy) and for one hospitalization due to Schönlein–Henoch purpura at the age of 4 years. He was also followed up for attention deficit/hyperactivity disorder during primary school. In the months leading up to our first visit, the patient had been referred to the emergency department for repeated instances of biliary and coffee-ground-like vomiting. Following these episodes, he started therapy with a PPI and prokinetic as well as a lactose-free diet without benefit. No evidence of previous severe acute respiratory syndrome coronavirus 2 infection or nonsteroidal anti-inflammatory drugs abuse was found, but he suffered a high level of stress related to school and family. Bowel habits tended toward constipation in the absence of reported stool alterations. Blood and fecal tests (including the search for *Helicobacter pylori* and parasites) proved negative save for increased C-reactive protein (CRP), and the abdominal ultrasound (US) revealed no pathological findings. Ten days after our first evaluation, we arranged for the execution of an upper gastrointestinal (GI) series in an outpatient setting. No evidence of gastroesophageal reflux disease (GERD) or hiatal hernia was found, but the stomach appeared oblong and replete with partially digested material even after prolonged fasting. Moreover, transit through the pylorus appeared to take a longer than average time, and the valve itself showed signs of mucosal thickening ( Fig. 1 ). These findings and the persistence of symptoms led us to admit the patient to our ward with a diagnosis of suspected inflammatory bowel disease (IBD) for further investigation. This diagnosis was a purely tentative one, mainly driven by his chronic abdominal pain and weight loss. First, we performed an esophagogastroduodenoscopy (EGD), showing marked gastric distension with hyperemic mucosa and weak peristalsis not transmitted to the pylorus. The latter showed signs of mucosal inflammation and was substenotic, the endoscopic instrument being barely able to pass through it. Biopsies were taken during this examination and subsequently revealed histological signs of aspecific gastritis and esophagitis. Following this unexpected finding, the patient was put on a nothing-by-mouth regimen with a nasogastric tube in place; support parenteral nutrition was undertaken via the peripheral vein. The following step was the execution of an and enteral magnetic resonance imaging, which showed an elongated pyloric canal (2 cm in length) with a global caliber of 2.4 cm considering all its layers in a longitudinal section a single muscular wall concentric thickness of 8 mm in the absence of other pathological findings. Extensive blood and fecal tests were performed again, including the search for HIV, hepatitis C virus, hepatitis B virus, hepatitis A virus, cytomegalovirus, Epstein-Barr virus, adenovirus, enterovirus, rotavirus, fungi, and *H. pylori* : all came out negative. Calprotectin and endocrinological markers were within limits, and autoimmunity tests showed antinuclear antibody pattern 1:60. At this point, 5 days after admission, multidisciplinary consultations with nutritionists and gastroenterologists took place. We decided to perform a balloon endoscopic pyloric dilation up to a caliber of 15 mm, which was performed on the same day: during the procedure, a nasojejunal tube was put in place and enteral immunomodulating nutrition was immediately undertaken starting with small amounts. A central venous line was also put in place to continue the supporting parenteral nutrition. A control abdominal US in the following days showed a pyloric canal with a maximum caliber of 16 mm with an overall wall thickness of 6 mm; jejunal nutrition was well tolerated. Following a significant reduction in gastric content output, the nasogastric tube was removed without the occurrence of vomiting in the following days. A subsequent dilation until a caliber of 20 mm was performed on day 13 from admission, after which the central venous line was removed, and parenteral nutrition was suspended. The patient was finally discharged after a hospital stay of 2 weeks with the recommendation of continuing PPI home therapy and enteral nutrition with a special formula. At an outpatient visit 2 weeks later, we found him in good clinical conditions, presenting without episodes of vomiting or epigastric pain and with a weight gain of 1.5 kg. We decided to increase the amount of enteral nutrition and start oral intake of clear fluids. EGD performed 1 month after discharge showed no anomalies; the pylorus was normally patent and without visible mucosal alterations. After consultations with our nutritionist, oral alimentation with selected soft foods was then resumed and well tolerated. We were able to remove the nasojejunal tube after 2 weeks at the following outpatient visit, and a free oral diet was initiated and tolerated. A final endoscopic control was performed 2 months and a half after the first discharge ( Fig. 2 ): no anomalies were found with a patent (if slightly rigid) pylorus, and biopsies taken found only minimal signs of aspecific gastric and duodenal chronic inflammation. At the last outpatient visit 6 months after the first discharge, clinical conditions were very satisfactory: the patient presented no further episodes of vomiting or epigastric pain, no GERD-like symptoms, weight gain, and a free diet. PPI therapy was discontinued.

## Discussion

As expected from the current literature about the etiology of NHIPS, 2 in our case, no definite causes were found. The patient's history characterized by chronic psychological stress does not allow us to exclude the possibility of a previous undiagnosed ulcer. Given the rarity of the condition, a possible pitfall of upper GI series and EGD is not considering NHIPS in the initial spectrum of feasible diagnosis. Our experience suggests that in cases of unexplained nature of chronic vomiting, EGD should be planned to provide for the possibility of performing immediate endoscopic dilation. The success of balloon dilation could be explained by allowing for a complete and longitudinal disruption of the seromuscular ring without any damage to mucosal integrity 3 (a result akin to that achieved through operative pyloromyotomy). In the available literature, resolution of the stenosis and the associated mucosal anomalies was achieved after three sessions as opposed to two in our case. 3 This could be explained by our choice to put a nasojejunal tube after the first dilation. Although the idea of positioning it right away during the first EGD was taken into consideration, we ultimately decided against it because the procedure was not undertaken in a fully equipped operating room and the device was not readily available, so we opted for rescheduling it to the following EGD. Enteral nutrition allows leaving the stomach to rest, reducing its dilated volume secondary to the NHIPS and regaining good motility. In our case, we decided to continue parenteral nutrition alongside it to bolster the nutritional status of the patient which was partially compromised after months of frequent vomiting and weight loss. In fact, enteral nutrition was administered in small amounts (with a gradual increase) in the first day, and it could not alone meet the patient's caloric and nutritional needs. In the cases where medical and/or endoscopic treatment had failed, 4 the last option could be the use of surgical procedures to resolve the condition. Pyloroplasty according to Heineke-Mikulicz (or variants thereof) appears to be the most employed intervention in the available literature. 4 In one article, antrectomy with gastroduodenal anastomosis has been described too. 2 However, it was performed in cases where acquired pyloric stenosis was a consequence of fibrosis due to ulceration, thus not meeting the requirements to be described as idiopathic. Since we achieved full dilation with an endoscopic approach, surgery was not taken into consideration for our patient. The multidisciplinary approach led a key role in the therapeutic planning of this case, with specialists providing valuable and specific insight, as for the nasojejunal tube, which contributed to successfully managing the case endoscopically and avoiding surgery. For comparison, Table 1 summarizes cases ascribable to NHIPS in the available literature and their diagnostic and therapeutic course, together with our experience. The search was conducted using the keywords “non-hypertrophic pyloric stenosis” and yielded a total of three articles collectively describing five cases. Interestingly, it appears that affected individuals are predominantly male, while ages at presentation show much greater variability. Only one other case of NHIPS was diagnosed in adolescence, but it was treated directly with surgery.

**Table 1:** Cases ascribable to NHIPS in the available literature with their diagnostic and therapeutic course, compared with our experience

Study	Age	Sex	Presentation	Symptoms duration	Exams	Biopsy	Intervention	Outcome
Hameury et al (2007)	15 y	F	NBV	Data not available	EGD	Mucosal inflammation	HMP	Cured
Hameury et al (2007)	2 y	M	NBV	Data not available	EGD	Not done	HMP after unknown number of ED	Cured
Hameury et al (2007)	17 mo	M	NBV	Data not available	EGD	Not done	HMP after unknown number of ED	Cured
Karnsakul et al (2010)	18 mo	M	NBV, FTT	3 wk	UGS, EGD, CT, X-ray, US	NAD	ED (three sessions, 2 wk intervals)	Cured
Ceccanti et al (2012)	6 y	M	NBV, abdominal pain, weight loss	1 y	X-ray, UGS, EGD, MRI	NAD (but suspect previous ulcer)	HMP after four ED sessions (2 mo intervals)	Cured
Ferlini et al (2022, present study)	15 y	M	NBV, abdominal pain, weight loss	3 mo	US, UGS, EGD, MRI	Aspecific gastritis esophagitis	ED (two sessions, 1 wk interval)	Cured

Abbreviations: CT, computed tomography; ED, endoscopic dilations; EGD, esophagogastroduodenoscopy; FTT, failure to thrive; HMP, Heineke–Mikulicz pyloroplasty; MRI, magnetic resonance imaging; NAD, no anomalies detected; NBV, nonbilious vomiting; NHIPS, nonhypertrophic idiopathic pyloric stenosis; UGS, upper gastrointestinal series; US, ultrasound.

## Conclusion

In conclusion, NHIPS in children is still a poorly understood medical entity, and evidence about the best course of action for its treatment is lacking. Based on our experience and the available literature, we propose considering the adoption of endoscopic pyloric dilation in association with enteral feeding through a nasojejunal tube as the first line of treatment before resorting to surgery. A multidisciplinary approach with nutritionists and gastroenterologists is extremely valuable for the resolution of similar cases.

## Notes

New Insights and the Importance for the Pediatric Surgeon Nonhypertrophic idiopathic pyloric stenosis is a rare entity in children, whose treatment is a matter of debate. We describe a case which we successfully treated with a combination of endoscopic dilations and jejunal feeding.
